# Influence of soil contamination with PAH on microbial community dynamics and expression level of genes responsible for biodegradation of PAH and production of rhamnolipids

**DOI:** 10.1007/s11356-016-7500-9

**Published:** 2016-09-01

**Authors:** Zuzanna Szczepaniak, Jakub Czarny, Justyna Staninska-Pięta, Piotr Lisiecki, Agnieszka Zgoła-Grześkowiak, Paweł Cyplik, Łukasz Chrzanowski, Łukasz Wolko, Roman Marecik, Wojciech Juzwa, Katarzyna Glazar, Agnieszka Piotrowska-Cyplik

**Affiliations:** 1 Institute of Food Technology of Plant Origin, Poznan University of Life Sciences, Wojska Polskiego 31, 60-624 Poznań, Poland; 2 Institute of Forensic Genetics, Al. Mickiewicza 3/4, 85-071 Bydgoszcz, Poland; 3 Department of Biotechnology and Food Microbiology, Poznan University of Life Sciences, Wojska Polskiego 48, 60-627 Poznań, Poland; 4 Faculty of Chemical Technology, Poznan University of Technology, Berdychowo 4, 60-965 Poznań, Poland; 5 Department of Biochemistry and Biotechnology, Poznan University of Life Sciences, Dojazd 11, 60-632 Poznań, Poland; 6 Department of Forest Technology, Poznan University of Life Sciences, 71C Wojska Polskiego St., 60-625 Poznan, Poland

**Keywords:** Bioaugmentation, Dioxygenase, MiSeq technology, PAHs, Rhamnolipids, Rhamnosylotransferase

## Abstract

The aim of this study was to evaluate the effect of bioaugmentation and addition of rhamnolipids on the biodegradation of PAHs in artificially contaminated soil, expression of genes crucial for the biodegradation process (PAHRHDαGN, PAHRHDαGP), and the synthesis of rhamnolipids as well as population changes in the soil bacterial metabiome. The positive effect of bioaugmentation and addition of rhamnolipids on the bioremediation of the majority of PAHs was confirmed during the early stages of treatment, especially in case of the most structurally complicated compounds. The results of metagenomic analysis indicated that the initial changes in the soil metabiome caused by bioaugmentation diminished after 3 months and that the community structure in treated soil was similar to control. The survival period of bacteria introduced into the soil via bioaugmentation reached a maximum of 3 months. The increased expression of genes observed after addition of PAH into the soil also returned to the initial conditions after 3 months.

## Introduction

The high interest in the bioremediation of polycyclic aromatic hydrocarbons is directly associated with their increasing occurrence in the environment related to civilization progress. Many PAHs exhibit toxic, carcinogenic, mutagenic and/or teratogenic properties; therefore, it is justified to search for effective methods to eliminate such compounds from the environment (Gupta et al. 2015).

Removal of organic pollutants, such as polycyclic aromatic hydrocarbons, from the environment is carried out using both physicochemical and biological methods. However, the latter are described in the literature as time-, energy-, and resource-saving as well as cost-effective (Haritash and Kaushik 2009). Bioremediation is one of the biological methods based on the use of microorganisms to decompose organic compounds. However, it has several limitations, especially in the case of water-insoluble hydrocarbons. The low bioavailability of hydrophobic compounds to microorganisms reduces their biodegradability. Generally, it is well known that in the case of biodegradation of mixtures, such as diesel oil, the aliphatic fraction is most susceptible to biological decomposition, while the fractions containing complex compounds (e.g., PAHs) are biotransformed at a relatively slow rate (Kostka et al. 2011; Pasumarthi et al. 2013).

For several years, scientists have been searching for methods which may increase the efficiency of bioremediation processes. Recently, much attention has been paid to strategies such as bioaugmentation or the addition of surfactants (Lladó et al. 2013; Szulc et al. 2014; Teng et al. 2010).

There are numerous literature reports which confirm the positive effect of surfactants on the biodegradation process. Researchers use both synthetic and natural surfactants, including biosurfactants, which are considered as a more eco-friendly alternative to chemical surface active agents (Sachdev and Cameotra 2013). According to Ławniczak et al. (2013), the stimulation of biodegradation by surfactants is associated with increased bioavailability of the substrates by mechanisms such as emulsification and solubilization. Moreover, the authors also mention the possible changes in cell surface properties of microorganisms, such as hydrophobicity or permeability. Additionally, the presence of surfactants may affect the oxygen content in contaminated soil, which in turn improves the conditions for growth and reproduction of microorganisms (Choi et al. 2009; Park et al. 2009). On the other hand, the inhibitory effect of surfactants on biodegradation, mainly associated with their toxicity (especially when used at high concentrations), is also observed. This results from the interaction of surfactants with the lipid components of the membrane, or the reaction with proteins necessary for proper functioning of cells (Ławniczak et al. 2013; Volkering et al. 1998).

However, the low abundance of microorganisms with appropriate metabolic capabilities to biotransform the pollutant at the contaminated site may result in low bioremediation efficiency, regardless of the use of surfactants in order to increase the bioavailability of the substrate. Many researchers suggest using bioaugmentation (defined as the addition of individual strains or microbial consortia with desired catabolic capabilities) in order to increase the biodegradation potential in the polluted area (Fantroussi and Agathos 2005; Silva et al. 2009). Owsianiak et al. (2009) emphasize that consortia isolated from contaminated sites are particularly valuable in terms of degradation of xenobiotics. On the other hand, Silva et al. (2009) reported no positive impact of bioaugmentation using individual fungi and bacteria as well as fungal consortia on the biodegradation of a mixture of PAHs. Such diverse results are associated with a multitude of biotic and abiotic factors which may affect the success of bioaugmentation.

Among the biotic factors, the competition between indigenous microorganisms and those introduced via bioaugmentation is mentioned most frequently (Mrozik and Piotrowska-Seget 2010). The adaption of the introduced microorganisms to the environmental conditions and maintenance of high metabolic activity seem to be key factors in the success of bioremediation. Groups of microorganisms present in the environment are characterized by a complexity of interrelationships which determine the biodegradation potential of the system (Zhang et al. 2006). Metagenomic analyses allow to determine the structure of the indigenous population and track its changes after bioaugmentation, due to the fact that they are culture-independent. Moreover, they also allow to conduct a specific diagnosis through qualitative evaluation of polymorphisms of genes which are crucial in terms of enzymatic degradation of contaminants.

The aim of this study was to define the effect of selected microorganisms, rhamnolipids and a combined approach (bioaugmentation + rhamnolipids) on the biodegradation of PAHs and changes of bacterial metabiome in PAH-contaminated soil. Metapopulation studies using NGS technologies (MiSeq Technology, Illumina) allowed to characterize the roles of different species in the microbial consortium and their functioning in the environment with autochthonous microorganisms. The expression of genes crucial for the biodegradation process (PAHRHDαGN, PAHRHDαGP) and the synthesis of rhamnolipids (RhlA, RhlC) as well as the metabolic activity of microorganisms, the efficiency of PAHs biodegradation and the rhamnolipids residues were determined. Analyses were carried out in soil systems for 365 days, after 0, 1, 3, 6, and 12 months of the experiment.

## Materials and methods

### Soil, microbial consortium, and rhamnolipids

The soil used in the experiments was collected from the center of Poznań, Poland (N 52.428674, E 16.900955). According to the soil texture analysis, it could be classified as silt loam. The soil had the following characteristics: sand 40 %, silt 52 %, clay 8 %, moisture 55 %, organic matter 16 %, pH = 6.4, and included the following main elements (mg*kg^−1^): Ca 18.6, Mg 8.3, N 598, P 1.2, K 24.0.

Microorganisms were added into the soil systems in the form of consortia derived from an area in the Polish Carpathian Mountains which is permanently contaminated by petroleum hydrocarbons. Prior to their introduction, the microorganisms had been cultivated for 5 days in 100-ml Duran-Schott bottles containing 250 ml of mineral medium (Cyplik et al. 2011). Diesel oil (2 %) was used as the sole carbon source.

A commercial solution of high-purity rhamnolipids from AGAE Technologies (USA) was used in the research.

### Chemicals

The following amounts of respective polycyclic aromatic hydrocarbons (Sigma Aldrich) were used for each sample during the experiments: fluorene 348.4 mg; phenanthrene 348.4 mg; anthracene 348.4 mg; pyrene 300 mg; fluoranthene 98.8 mg; acenaphthylene 98.8 mg; acenaphthen 98.8 mg; chrysene 4 mg; benzo[a]anthracene 4 mg; benzo[b]fluoranthene4 mg; benzo[k]fluoranthene4 mg; benzo[a]pyrene 4 mg.

### Experimental variants

The study concerning the biodegradation of PAHs was carried out under natural conditions in containers (volume of 4 L) filled with 2 kg of soil (with a geofiber-based bottom in order to allow the runoff water to freely percolate into the deeper soil layers). The geofiber surface at the bottom of each container was covered with a layer of activated carbon and an additional layer of geofiber. This ensured that there was no direct contact between the soil and activated carbon layer and enabled the evaluation of analyte loss. Appropriate amount of each PAH was dissolved in ethyl acetate and the solution was added to 200 g of the soil sample. Upon evaporation of ethyl acetate, the soil was used as a PAH carrier, which was introduced into natural soil (Szczepaniak et al. 2015).

The following experimental systems were studied: (i) 2 kg of soil (reference sample); (ii) 2 kg of soil + polycyclic aromatic hydrocarbons; (iii) 2 kg of soil + polycyclic aromatic hydrocarbons + additional microbial consortium; (iv) 2 kg of soil + polycyclic aromatic hydrocarbons + rhamnolipids; (v) 2 kg soil + polycyclic aromatic hydrocarbons + additional microbial consortium + rhamnolipids.

The experiment was conducted in containers, which were placed in soil in order to ensure maximum imitation of natural conditions. The experiment was started in March 2014 and it was conducted during a 1-year period. The temperature was not regulated. Six subsamples per plot were collected with a hand auger and mixed to yield one mean sample per plot. The soil samples were directly sieved to 1 mm and parts of the samples were used for chemical analyses.

Analyses were carried out (i) at the beginning and after: (ii) 1 month; (iii) 3 months; (iv) 6 months; (v) 12 months of treatment.

### Analyses of PAH biodegradation

#### Preparation of samples for GC-MS analyses

In order to determine the dissipation of PAHs after 0, 1, 3, 6, and 12 months, one replicate of each setup was sacrificed for extraction and analysis. The samples were prepared in the following manner: First, an aliquot of deuter-labeled internal standards (acenaphthene-d10, anthracene-d10 and chrysene-d12) was added; next, 80 ml of acetone were added to the samples, which were shaken on a vortex mixer and placed into ultrasonic bath. After 10 min, the samples were shaken vigorously one more time to mix the sample matrix on the bottom of the flask, sonicated for another 10 min and was shaken for 20 min at 250 rpm on a horizontal shaker. Then, 20 g of anhydrous MgSO_4_ were added into the samples and the procedure of sonication and mechanical shaking was repeated as described earlier. Finally, 40 ml of hexane were added and the procedure of sonication and mechanical shaking was repeated once again.

After the extraction, 20 ml of the obtained extract were collected into a vial and subjected further to clean-up procedure. Firstly, acetone was removed. For this purpose, a 2.5 ml aliquot of the extract was shaken with 10 ml of 10 mM NaOH and 0.1 M of NaCl. After phase separation, 100 μl of the hexane phase were applied on the Florisil column. The analytes were eluted with a 400 μl portion of hexane/MTBE mixture (1:3 *v*/*v*). Finally, an aliquot of 2-ethylnaphthalene solution (serving for recovery control) was added to the collected eluate and the samples were subjected to gas chromatographic analysis.

#### GC-MS parameters

The GC-MS analyses were performed on a Shimadzu 17A chromatograph coupled with a 9200 mass spectrometer. The samples were injected at the temperature of 250 °C and a ratio of 1:20. The analytes were separated on a RTX-5 column (30 m length, 0.25 mm internal diameter and 0.25 μm film thickness), using helium as the carrier gas (flow rate of 1 ml/min). The temperature program was as follows: initial temperature of 50 °C held for 1 min, ramped to 270 °C at 15 °C/min, and then ramped to 300 °C at 5 °C/min. The MS operated in SIM mode with ion source temperature of 250 °C and ion source voltage of 70 eV, solvent delay 290 s and acquisition rate 10 spectra.

### Analyses of rhamnolipid residuals

#### Preparation of samples for HPLC analyses

For determination of rhamnolipids, 20 g of dried soil was subjected to a four step ultrasound-assisted extraction with ethanol. The obtained extract (50 ml) was filtered through 0.45 μm pore-size PTFE syringe filter, diluted with a water to acetonitrile mixture (1:4 *v*/*v*) and subjected to HPLC-MS/MS analysis. The extraction and analysis were performed in three replicates for each microcosm.

#### HPLC parameters

The HPLC-MS analysis was carried out using the UltiMate 3000 RSLC chromatographic system from Dionex (Sunnyvale, CA, USA). Samples (5 μL) were injected into a Gemini-NX C18 column (100 mm 2.0 mm I.D.; 3 μm) from Phenomenex (Torrance, CA, USA). The mobile phase used for the analysis consisted of 5 mmol l^−1^ ammonium acetate in water and acetonitrile at a flow rate of 0.3 ml min^−1^. The following gradient was used: 0 min 55 %; 4 min 55 %; 4.5 min 90 %; and 6 min 90 % of acetonitrile. The LC column effluent was directed to the API 4000 QTRAP triple quadrupole mass spectrometer from AB Sciex (Foster City, CA, USA) through the electrospray ionization source (Turbo Ion Spray). The ionization source operated in negative ion mode. The dwell time for each mass transition detected in the MS/MS multiple reaction monitoring mode was set to 20 ms. All rhamnolipids were detected using the following settings for the ion source and mass spectrometer: curtain gas 10 psi, nebulizer gas 40 psi, auxiliary gas 45 psi, temperature 450 °C, ion spray voltage −3500 V, and collision gas set to medium. The declustering potential was set to −80 V for monorhamnolipids and −85 V for dirhamnolipids. The collision energy for quantitative transitions was set to −28 V for monorhamnolipids and −35 V for dirhamnolipids. The collision energy for confirmatory transitions was set to −21 V for monorhamnolipids and −29 V for dirhamnolipids.

### Determination of the half-lives of PAHs and rhamnolipids

The half-lives of PAHs and rhamnolipids were calculated using a single, three-parameter equation (exponential decay).

### Flow cytometry analyses

In order to determine the metabolic activity of microorganisms during the experiment, flow cytometry analyses were conducted. Samples from soil microcosms (10 g) were washed with distilled water (100 ml) and shaken on the MS3 vortex (Ika, Germany) for 5 min at 2500 rpm. To avoid binding of fluorescent dyes to non-cellular particle, 2 ml of the suspension was filtered through a filter with a 20-m nylon net syringe filter (Merck Millipore, Germany). Then, all the samples were centrifuged at 4000 rpm for 6 min (Heraeusbiofuge primo R), the supernatant was decanted, and the precipitate was washed with 0.5 ml PBS. Green reagent (1.5 l) and propidium iodide (0.8 l) were added and the samples were incubated at 38 °C for 10 min. Green reagent is a fluorescent dye for measuring redox, while propidium iodide is used to measure cell viability on the basis of intensive incorporation into cells with damaged cell wall and lack of penetration (and hence the absence of dying) in intact cells. The analyses on flow cytometer were performed during the last step.

The redox potential of microbial cells and viability assessment were evaluated using the BacLight™ Redox Sensor™ Green Vitality Kit by Invitrogen. Samples were analyzed using a BD FACS Aria™III (Becton Dickinson, USA) flow cytometer (cell sorter). The populations were defined by gating in the dot plots of green fluorescence (FITC-A) versus red fluorescence (PE-Texas Red-A). Each sample was analyzed in triplicate. The estimation of cellular redox potential was performed using medians of green fluorescence (FITC-A) signals of gated populations defined on a bivariate dot plot (FITC-A vs. PE-Texas Red-A) (Szczepaniak et al. 2015).

### Identification of microorganisms

#### DNA extraction

Total DNA was extracted from 500 mg of each soil sample using Genomic Mini AX Soil kit (A&A Biotechnology) according to manufacturer’s instruction. The extracted DNA was quantified using Quant-iT HS ds.-DNA assay kit (Invitrogen) on Qubit2 fluorometer; 2 μl of extracts were examined on a 0.8 % agarose gel.

#### PCR amplification

Region IV of bacterial 16S RNA gene was amplified using universal primers 515F and 806R: containing reverse complement of 3′ Illumina adapter, golay barcode, reverse primer pad, reverse primer linker, and reverse primer (Table 1). Genomic DNA (100 ng) was used for PCR amplification in 50 μl volume in reaction containing the following: 1× PCR reaction mix, 0.25 μM each primer, and 5 U Taq DNA polymerase (A&A Biotechnology). Following conditions were used for PCR amplification: initial denaturation 95 °C for 3 min; 35 cycles of denaturation 30 s at 94 °C, annealing 30 s at 52 °C and extension 2 min at 72 °C and a final extension at 72 °C for 10 min. Products were purified in Clean-Up columns (A&A Biotechnology) according to manufacturer’s protocol. The libraries were constructed from amplicons using NEBNext® DNA Library Prep Master Mix Set for Illumina (New England Biolabs UK). Then, the libraries were polled at equimolar concentration. Pooled library was quantified using a Qubit Fluorometer and dsDNA HS assay kit (Life Technologies, USA). The library was denatured with 0.2 N NaOH and diluted with HT1 buffer (Illumina, USA) to a final concentration of 8 pM. To balance the overall lack of sequence diversity, a spike-in of denatured Phix was added to the concentration of 40 %. Sequencing was conducted on an Illumina MiSeq (Illumina, USA) using paired-end (2 × 250) MiSeq Reagent Kits v2 (Illumina, USA). Three sequencing primers were based on Caporaso et al. 2012 (Table 1). The sequencing reaction was performed with MiSeq Illumina instrument and MiSeq Reagent Kit v2 (2 × 250 bp).

**Table 1:** Characteristics of primer for PCR amplification, sequencing (MiSeq Technology) and real-time PCR

Primers	Sequence (5′ to 3′)	Reference
PCR amplification		
Forward 515F	AATGATACGGCGACCACCGAGATCTACACTATGGTAATTGTGTGCCAGCMGCCGCGGTAA^1^	Caporaso et al. (2012)
Reverse 806R	CAAGCAGAAGACGGCATACGAGATXXXXXXAGTCAGTCAGCCGGACTACHVGGGTWTCTAAT^2^	
Sequencing		
Read 1	(TATGGTAATT)^6a^ (GT)^7a^ (GTGCCAGCMGCCGCGGTAA)^8a^	
Read 2	(AGTCAGTCAG)^6b^ (CC)^7b^ (GGACTACHVGGGTWTCTAAT)^8b^	
Index	(ATTAGAWACCCBDGTAGTCC)^6c^ (GG)^7c^ (CTGACTGACT)^8c^	
RT-PCR		Schmidberger et al. 2013
RhlA		
Forward	F 5′-GATCGAGCTGGACGACAAGTC-3′	
Reverse	R 5-GCTGATGGTTGCTGGCTTTC-3′	
RhlC		
Forward	F 5′-ATCCATCTCGACGGACTGAC-3′	
Reverse	R 5′-GTCCACGTGGTCGATGAAC-3′	
PAH-RHDα GN		Cébron et al. 2008
Forward	GAG ATG CAT ACC ACG TKG GTT GGA	
Reverse	AGC TGT TGT TCG GGA AGA YWG TGC MGT T	
PAH-RHDα GP		
Forward	CGG CGC CGA CAA YTT YGT NGG	
Reverse	GGG GAA CAC GGT GCC RTG DAT RAA	
16 S rDNA		
968 Forward	AAC GCG AAG AAC CTT AC	
1401 Reverse	CGG TGT GTA CAA GAC CC	

^1^Containing 5′ Illumina adapter, forward primer pad, forward primer linker, and forward primer sequence

^2^Containing reverse complement of 3′ Illumina adapter, golay barcode, reverse primer pad, reverse primer linker, and reverse primer

^6a^forward primer pad

^6b^reverse primer pad

^6c^reverse complement of reverse primer

^7a^forward primer linker

^7b^reverse primer linker

^7c^reverse complement of reverse primer linker

^8a^forward primer

^8b^reverse primer

^8c^reverse complement of reverse primer pad

#### Bioinformatic analysis

The sequencing data was processed using CLC Genomic Workbench 8.5 and CLC Microbial Genomics Module 1.2. (Qiagen, USA). Total number of reads ranged from 438,216 to 461,123. After sequencing, the reads were demultiplexed to the probes and the overlapping paired-end reads were merged (70 % of total reads) and trimmed to yield fragments of 291 nt. Just fragments which passed the merging were retained for downstream processing. Chimeric reads (from 27,121 to 29,785) were filtered and remaining sequences were assigned to operational taxonomic units (OTUs). Number of reads which passed merging and trimming ranged from 136,576 to 154,294. Reads were clustered against the SILVA v119 99 % 16S rRNA gene database (July 24, 2014, Quast et al. 2013). Rarefaction analysis with a depth of 80,000 sequences per sample was used to calculate alpha diversity measured using OTU abundance, Shannon’s index, Chao 1 bias-corrected, and phylogenetic diversity.

### RT-PCR

Total RNA was extracted from 1 g of soil samples by RNA PowerSoil Total RNA Isolation Kit (MO BIO Laboratories, Inc.). Genomic DNA was digested using TURBO DNA-free Kit (Ambion by Life Technologies). RNA concentration was measured using Qubit RNA HS Assay Kit on Qubit Fluorometer (Invitrogen/Life Technologies). Random-primed cDNA synthesis was conducted using High-Capacity cDNA Reverse Transcription Kit and MultiScribeMuLV Reverse Transcriptase. Gene expression was analyzed using a Power SYBR Green PCR Master Mix (Life Technologies) on ABI 7500 SDS (Applied Biosystems). Primers used for real-time PCR are listed in Table 1. Total bacterial RNA was quantitated by real-time PCR amplification of fragment of bacterial 16S ribosomal RNA with universal bacterial primers and TaqMan MGB probe using TaqMan Universal Master Mix II (Life Technologies) on ABI 7500 SDS (Applied Biosystems). Sequences of primers and probe used are listed in Table 1. All analysis was done in triplicates. In order to compare the gene expression in each sample, the mean expression index was calculated according to formula: C_T_ target/C_T_ 16S using data from three analyses. This parameter reflects the expression level of a specific gene compared to the expression level of the universal gene (16S RNA) in the whole metabiome.

## Results and discussion

### PAH biodegradation

GC-MS analyses were performed in order to determine the biodegradability of the individual PAHs. The results indicated a close correlation between the structure of the compounds and the rate of biodegradation. Calculated half-lives of PAHs showed that the increase in the number of rings resulted in the increase of half-lives of the compounds from 0.2 months for the simplest compounds to 14 months in the case of pentacyclic hydrocarbons. In all cases (except acenaphthene and acenaphthylene), the half-lives were shorter in variants containing additional microorganisms or rhamnolipids and the combined method (bioaugmentation + rhamnolipids). However, no differences between the mentioned variants were observed in general. In the case of the combined method, it was not possible to calculate the half-lives for three PAHs (benz[b]fluoranthene, benz[k]fluoranthene, benzo[a]pyrene), since the residues accounted for more than half of the initial mass (Table 2). The results which indicate the increase of half-lives as a result of increased structural complexity of the compounds are consistent with those obtained by Silva et al. (2009). The researchers noted that the LMW-PAHs (naphthalene, phenanthrene, and anthracene) and the HMW-PAH (pyrene) were removed more rapidly compared to the HMW-PAHs (benz[a]anthracene and benz[a]pyrene).

**Table 2:**
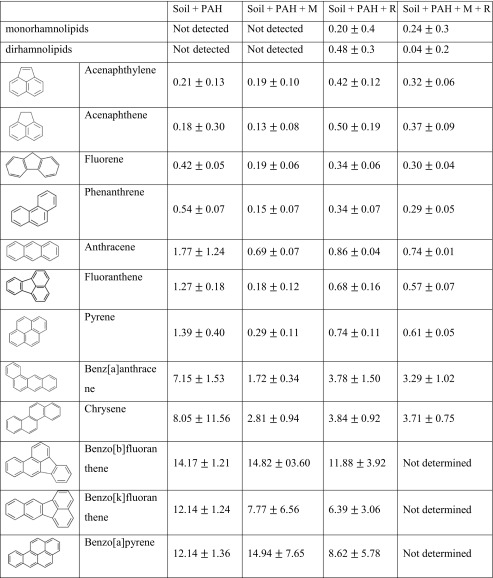
Half-lives in months of studied PAHs and rhamnolipids

**Fig. 1:**
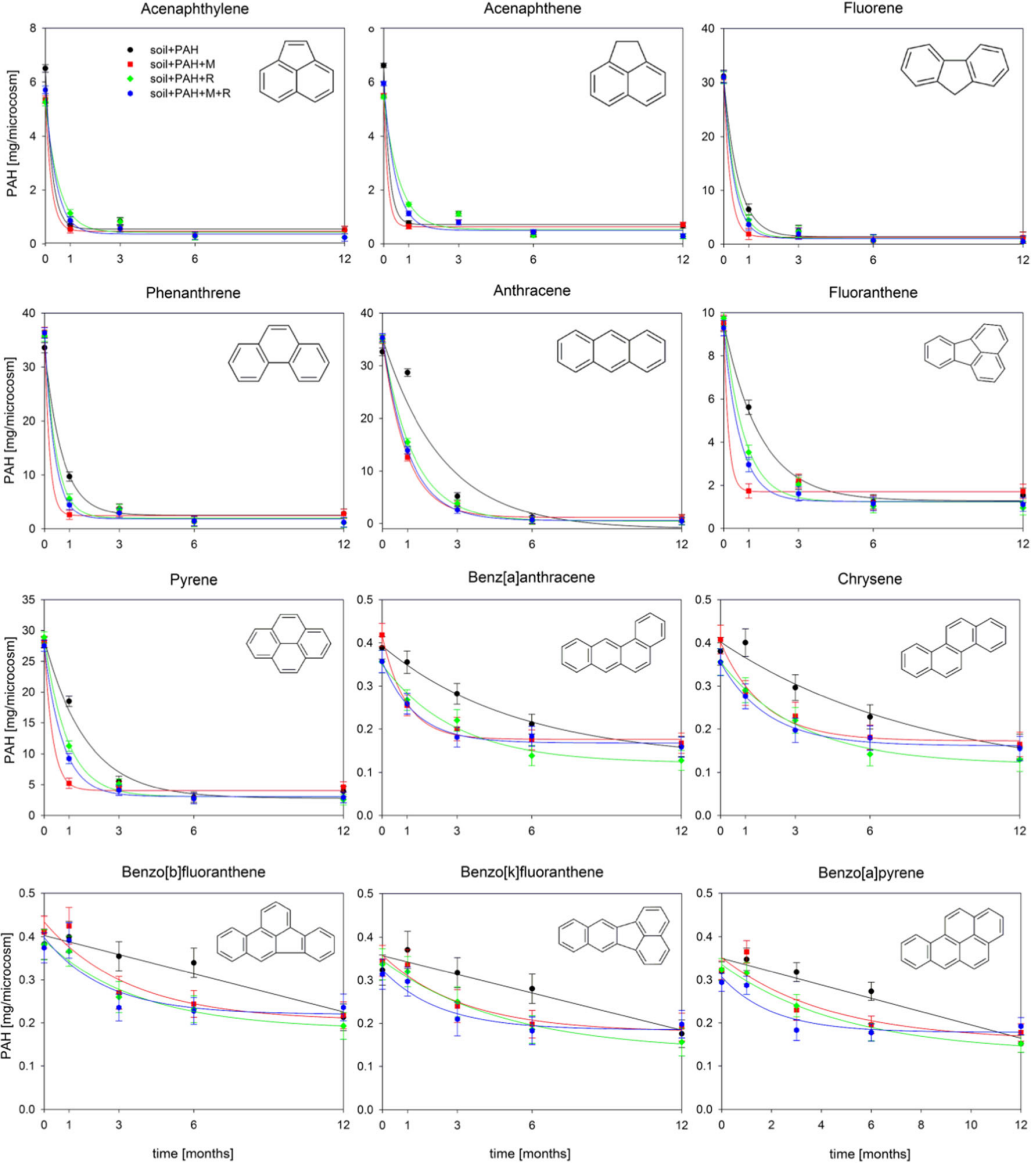
Biodegradation of polycyclic aromatic hydrocarbons during 12 months of process

The obtained results indicated that bioaugmentation, addition of rhamnolipids and the use of the combined approach (bioaugmentation + rhamnolipids) had a positive effect on the biodegradation efficiency compared to natural attenuation during the early stages of the process (with the exception of acenaphthylene and acenaphthene), especially for the most structurally complex compounds. However, no differences in PAHs content in soil were observed between the natural attenuation and other variants after 12 months of treatment. Moreover, the time needed to equate natural attenuation efficiency with the other treatments methods varied depending on the molecular structure of PAHs (from 1 to 3 months for tricyclic hydrocarbons up to 12 months for pentacyclic compounds). The obtained results were presented in Fig. 1.

**Fig. 2:**
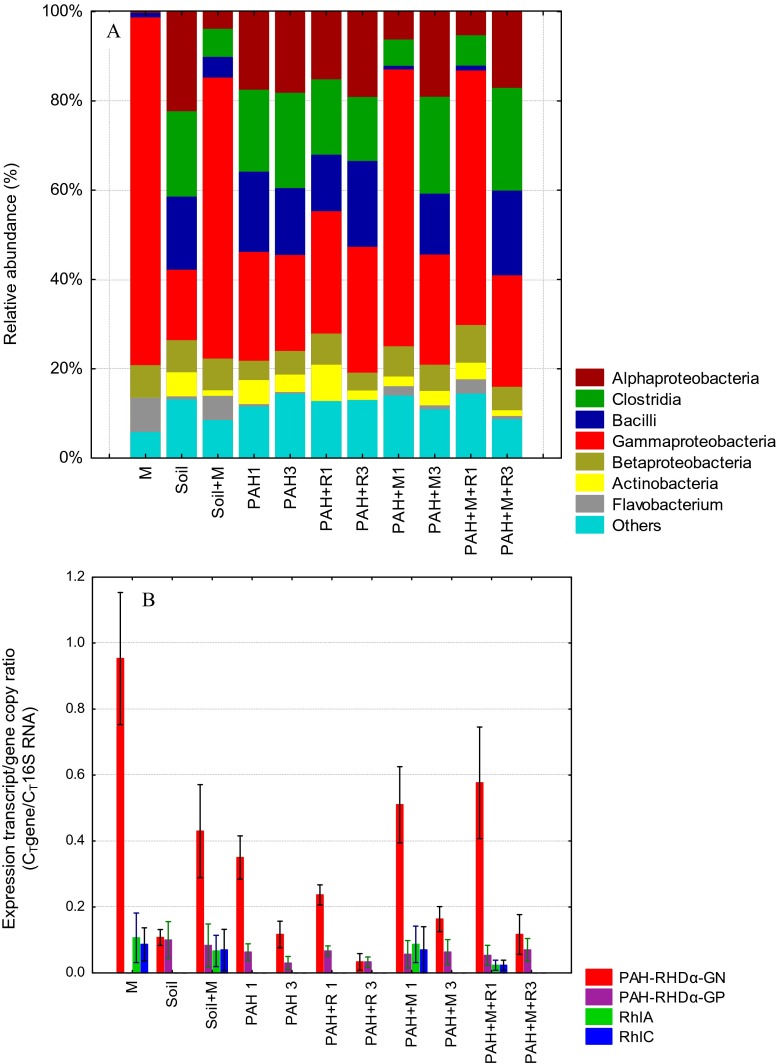
Relative abundance of bacterial classes (**a**) and quantitative changes in RhlA, RhlC, PAHRHDαGN, and PAHRHDαGP expression level genes (**b**) present in the soil after 1 and 3 months of biodegradation

In order to identify the bacterial consortium (M), a metagenomic analysis of the gene encoding 16S rRNA was conducted on the basis of V4 hypervariable region of the 16S rRNA gene (Fig. 2a). The use of next generation sequencing allowed to identify the bacterial consortium (M) as members of seven classes, with the dominance of *Gammaproteobacteria* (77.9 %), *Betaproteobacteria* (7.7 %), and *Flavobacteria* (7.3 %). Three genera dominated among *Gammaproteobacteria*: *Pseudomonas* (58 %), *Achromobacter* (14 %), and *Stenotrophomonas* (12 %). On the other hand, the results of the 16S metagenomic analysis of the soil metabiome indicated that the soil was more diverse. A total of 57 classes were identified in the bacterial microbiome, with the dominance of *Alphaproteobacteria* (22.5 %), *Clostridia* (19.1 %), *Bacilli* (16.4), *Gammaproteobacteria* (15.8 %), *Betaproteobacteria* (7.2 %), and *Actinobacteria* (5.4 %).

The contamination of soil with PAHs caused significant changes in the soil bacterial community. The dominance of species belonging to *Gammaproteobacteria* could be observed after the first month of biodegradation. Their percentage share in the population amounted to 24.4 % after the first month and did not change significantly (*p* > 0.05) until the third month of the process. The remaining dominant classes included *Clostridia* (21.36 %), *Alphaproteobacteria* (18.3 %), and *Bacilli* (15 %). Similar changes in the community structure were observed after the addition of rhamnolipids. In this case, the *Gammaproteobacteria* also dominated the system (28.2 %) and the share of remaining classes (*Alphaproteobacteria*, *Clostridia*, and *Bacilli*) ranged from 14.3 to 19.3 %. Notable differences in the community structure were observed in case of PAH-contaminated soil with the addition of the microbial consortium (PAH + M). A significant increase of the abundance of bacteria added in the form of a preparation (PAH + M) could be observed in the soil metabiome. *Gammaproteobacteria* was the dominant class, which accounted for 57 to 62 % of the total bacterial population. The abundance of the remaining identified classes (*Alphaproteobacteria*, *Clostridia*, *Bacilli*, *Betaproteobacteria*, and *Actinobacteria*) ranged from 0.8 to 8.4 %. However, the community structure returned to the initial state (the structure determined for control soil) after 3 months of the biodegradation process.

Attachment of oxygen to the aromatic ring catalyzed by dioxygenases (PAHRHDα) is the key step for biodegradation of PAH, which results in the formation of *cis*-dihydrodiol. The enzyme catalyzing this reaction (dioxygenase) is composed of a large α subunit and a smaller β subunit. The α subunit contains a region named [Fe2-S2] Rieske center. This center was found in numerous enzymes with a similar catabolic activity to PAHRHDα but with different substrate specificity. The presence of various genes encoding similar enzymes among Gram positive as well as Gram negative bacteria marks the significance of the presence of both these groups for a proper assessment of biodegradation efficiency among the whole microbial consortium (Cébron et al. 2008). An indirect method was employed in order to determine the expression of all the studied genes, which was based on the determination of the number of cycles for the analyzed gene (C_Tgene_) and its comparison with an internal standard (the number of cycles for the gene encoding the 16S rDNA subunit—C_T16S_). The employed method allowed to establish the relative changes in the expression of genes with respect to the abundance of bacteria in soil. The determined C_T16S_ value for the 16S RNA gene (internal standard) ranged from 5.7 to 7.4 between the samples. During the first month of biodegradation, the expression of gene encoding dioxygenases in Gram negative bacteria (PAH-RHDα GN) was highest in soil with the addition of both rhamnolipids and the bacterial consortium (PAH + M + R) as well as in bioaugmented soil (PAH + M). The relative expression of these genes reached 0.723 ± 0.245 and 0.693 ± 0.194, accordingly, and these values were not statistically different (*p* > 0.05). On the other hand, the expression in untreated soil as well as soil with the addition of rhamnolipids (PAH + R) was lower and ranged from 0.365 ± 0.178 to 0.395 ± 0.087. These values were statistically different compared to bioaugmented soil (*p* < 0.05). After 3 months, the expression returned to the level observed in control soil. In contrast, the expression of gene encoding dioxygenases in Gram positive bacteria (PAH-RHDα GP) did not change significantly and ranged from 0.092 ± 0.037 to 0.120 ± 0.072.

The expression of genes encoding rhamnosyltransferase I (RhlA) and III (RhlC) was observed only in case of soils subjected to bioaugmentation (PAH + M and PAH + M + R); however, it was inhibited after the third month. No expression of genes associated with the biosynthesis of rhamnolipids was observed in case of control soil and soil with the addition of rhamnolipids (PAH + R) (Fig. 2b). Therefore, the presence of rhamnosyltransferases in soil was associated with their presence in the bacterial consortium, which was introduced to the soil. It is worth noticing that the addition of rhamnolipids did not inhibit the expression of RhlA and RhlC in the environment, and its decrease was associated with the decreased abundance of bacteria introduced into the soil via bioaugmentation (M).

**Fig. 3:**
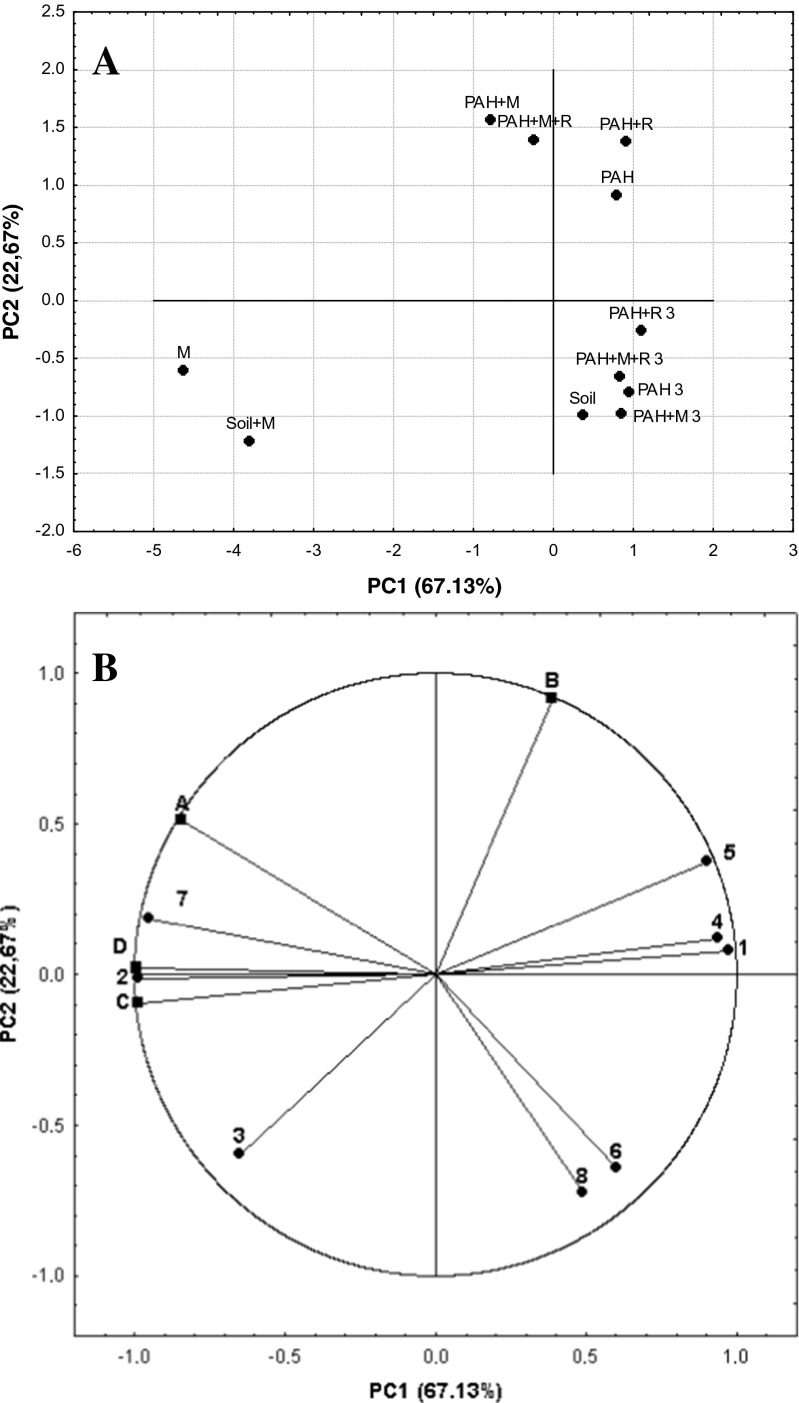
Principal component  analysis (PCA) of taxonomy profile (**a**) and correlations between relative abundance of bacterial classes and genes: RhlA, RhlC, PAHRHDαGN, PAHRHDαGP expression (**b**) during PAHs biodegradation

Principal component analysis (PCA) was employed in order to evaluate the changes in the soil bacterial metabiome. The results which reflect the grouping of the analyzed bacterial metabiomes are shown in Fig. 3a. The two first principal components are crucial and describe the variability of the initial data in 89.9 %. The first primary component carries 67.13 % of the data regarding microbial populations contained in the initial variables. It includes the positively correlated *Bacilli*, *Clostridia*, and *Alphaproteobacteria* as well as negatively correlated *Betaproteobacteria*, *Flavobacterium*, and *Gammaproteobacteria*. The second component is mainly associated with the presence of *Actinobacteria* and others and describes the variability of the analyzed data in 22.67 % (Fig. 3b). The high diversity of the soil bacterial microbiome and the introduced bacterial consortium (M) is also visible in Fig. 3a. The contamination of soil with PAHs as well as the addition of the selected microbial consortium and rhamnolipids caused significant changes in the soil metabiome, which are reflected as a visible cluster in the upper-right corner of the figure. However, after 3 months, the metabiomes were similar in terms of structure to control soil.

Analysis of mutual relationships between the specific classes of microorganisms and the expression of genes suggests a strong correlation between the expression of genes associated with the synthesis of rhamnolipids (RhlA and RhlC) and bacteria belonging to the *Gammaproteobacteria* class (which includes the *Pseudomonas* genus). A weak correlation between these genes and the expression of dioxygenases in Gram negative bacteria was established, whereas no correlation was found in Gram positive bacteria. The PCA analysis confirmed the positive correlation of dioxygenase G+ with *Bacilli* and *Clostridium* classes as well as dioxygenase G− with Gram negative bacteria belonging to *Betaproteobacteria*, *Flavobacterium*, and *Gammaproteobacteria*.

The alpha diversity indicators determined after 3 months of biodegradation did not differ significantly, which confirms the high potential of autochthonic microorganisms to remove the contaminants from soil via natural attenuation and to recover the initial community structure (Table 3).

**Table 3:** Alpha diversity measured using number of OTUs, Shannon’s index, Chao 1 bias-corrected, and phylogenetic diversity

	Soil	Soil + PAH	Soil + PAH + R	Soil + PAH + M	Soil + PAH + M + R
OTU observed	1984 ± 41	1930 ± 48	1956 ± 54	1990 ± 43	2014 ± 61
Shannon’s index	5.7 ± 0.2	5.4 ± 0.3	5.5 ± 0.2	5.5 ± 0.2	5.6 ± 0,3
Chao 1 bias-corrected	1464 ± 251	1432 ± 279	1451 ± 197	1487 ± 211	1496 ± 173
Phylogenetic diversity	4.89 ± 0.34	4.76 ± 0.28	4.95 ± 0.39	4.68 ± 0.19	4.79 ± 0.29

The use of bioaugmentation for biodegradation of PAHs has been described in detail by many authors (Fantroussi and Agathos 2005; Fernández-Luqueño et al. 2011; Mohan et al. 2006). Positive results were obtained using single strains (Colombo et al. 2011; Teng et al. 2010), as well as microbial consortia (Owsianiak et al. 2009). The bioaugmentation strategy turned out to be useful not only in the case of PAH-contaminated soil but also for diesel oil pollution (Bento et al. 2005; Szulc et al. 2014).

On the other hand, Silva et al. (2009) did not observe a positive impact of bioaugmentation in case of low molecular weight (LMW) and high molecular weight (HMW) PAHs, using both individual fungi as well as bacterial and fungal consortia, with the exception of *Aspergillus sp*. Similar results regarding no positive effect of the introduction of additional microorganisms on biodegradation were also observed by other researchers (Launen et al. 2002; Saponaro et al. 2002).

The obtained results indicated the positive effect of the use of biosurfactants in the early stages of the experiment (with the exception of acenaphthene and acenaphthylene). The ability to stimulate the biodegradation of PAHs by the use of surfactants has been confirmed by numerous researchers and is associated, e.g., with the mechanisms of increasing the bioavailability of hydrophobic compounds by their emulsification and/or solubilization, or altering the properties of the microorganisms cell surfaces (Ławniczak et al. 2013). Rodrigues et al. (2013) have reported that low concentrations of surfactants not only stimulated the decomposition of anthracene and fluoranthene but also indicated that the effect varied depending on the type and concentration of surfactant and the type of PAHs. An exceedingly high concentration or the wrong choice of surfactant can be toxic toward to microorganisms cells, resulting in a decrease of their number and, consequently, lower efficiency of the biodegradation process. Moreover, despite the natural origin of rhamnolipids, it is necessary to consider the need to select the optimal concentration for terrestrial environment. There are literature reports which indicate the possibility of decreasing of plant biomass (Millioli et al. 2009) and even increasing the toxicity of contaminants such as diesel oil by increasing their bioavailability in the presence of rhamnolipids (Marecik et al. 2012).

We hypothesized that the combined treatment method (which involved bioaugmentation and addition of rhamnolipids) would improve the biodegradation due to the advantages of both techniques and also solve the problem of inoculation/introduction of microorganisms into the soil. According to Mrozik and Piotrowska-Seget (2010), the use of surfactants and foams can facilitate the dispersion of inoculants into subsurface environment. However, the obtained results did not confirm this theory since no differences between the studied treatment methods (bioaugmentation, addition of rhamnolipids, and combined method) were observed. Similarly, Szulc et al. (2014) did not observe a significant positive impact of the combined approach compared to other strategies during research regarding biodegradation of diesel oil using bioaugmentation, biosurfactants, and the combined approach.

The obtained results indicated that it is reasonable to use bioremediation strategies such as bioaugmentation or rhamnolipid addition, when rapid purification of the area is necessary. According to Forsyth et al. (1995), the use of bioaugmentation is justified in the case of low or non-detectable amount of microorganisms that are able to degrade contaminants, in fields contaminated with compounds requiring multi-process remediation (e.g., a toxic to microbes), and for small-scale areas. No difference between the treatment methods after 12 months of experiment suggested that in the absence of time pressure, the introduction of additional microorganisms or surfactants may not be justified due to a sufficient number of contaminant-degrading microorganisms and possible natural attenuation. This is in agreement with the results obtained by Mesbaiah et al. (2014). The strain used in the studies was able to degrade the LMW PAHs without additional intervention. Moreover, Silva et al. (2009) clearly demonstrated the possibility of immediate reaction of native microorganisms to the addition of PAH and their ability to degrade both LMW and HMW added to soil.

### Rhamnolipid residues

The mixture of rhamnolipids used in the experiment contained monorhamnolipids and dirhamnolipids. Analyses were performed for variants with addition of rhamnolipids (soil + PAH + R; soil + PAH + M + R) as well as for the variants without rhamnolipids (soil + PAH; soil + PAH + M) in order to determine whether microorganisms in the studied soil were able to produce biosurfactants.

The obtained results demonstrated that monorhamnolipids dominated over dirhamnolipids in the solution of biosurfactants used in the experiment, which corresponded to the initial content of rhamnolipids in variants with their addition. In case of variants without biosurfactants addition, their contents for all treatment time remained at the background level. This suggested that microorganisms in studied systems did not produce rhamnolipids.

**Fig. 4:**
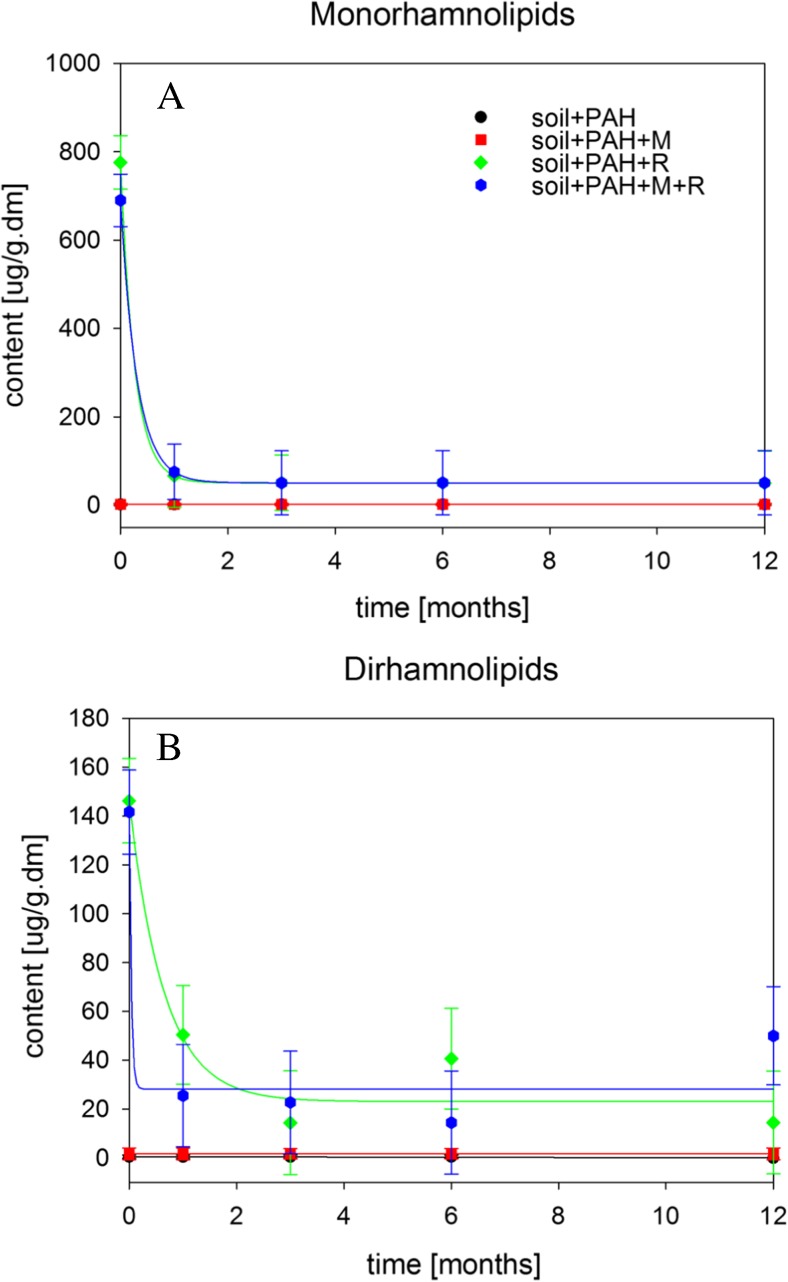
Biodegradation of monorhamnolipids (**a**) and dirhamnolipids (**b**) during 12 months of process

In addition, the analyses carried out after the first month of treatment proved that during the early stage of the experiment (up to the first month) rhamnolipids were almost entirely biodegraded. Similar results of rapid biodegradation of rhamnolipids were obtained by Szulc et al. (2014). During research regarding the effects of addition of rhamnolipids on the bioremediation of diesel-oil contaminated soil, the authors established that rhamnolipids should be reintroduced after 30 days (Fig. 4).

The increasing use of biosurfactants in many industries as well as in environmental protection is associated with, e.g., their natural origin resulting in their relatively low toxicity and biodegradability and therefore greater environmental benefits (Sachdev and Cameotra 2013). Natural surfactants can be successfully used as alternatives to synthetic surfactants. Singh and Cameotra (2014) suggested that the use of rhamnolipids contributed to higher effectiveness compared to Triton X-100 during biodegradation of atrazine herbicide by A6, belonging to the genus *Acinetobacter*.

The half-life of rhamnolipids was shorter compared to most of the PAHs in variants with the addition of biosurfactants. This means that rhamnolipids may not affect the bioavailability of PAHs in the later stages of bioremediation.

### Metabolic activity of microorganisms

**Fig. 5:**
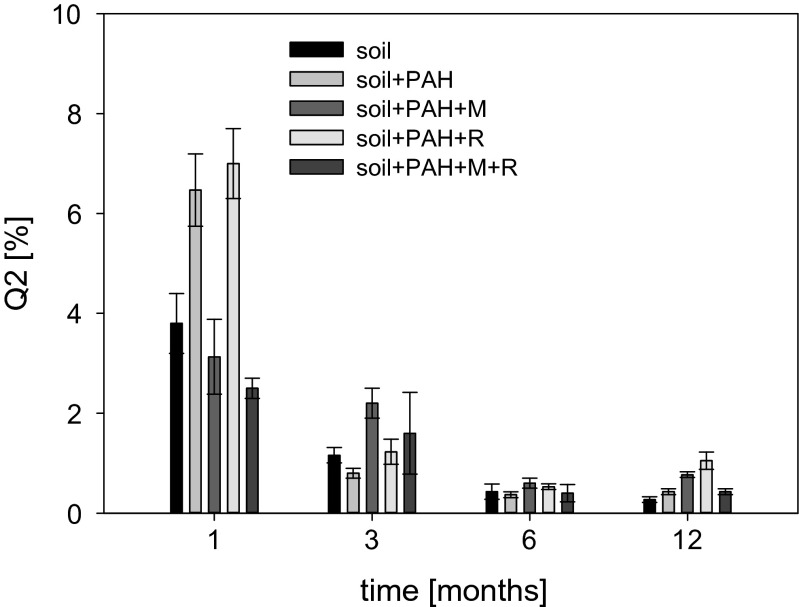
The percentage of the most metabolically active microorganisms (%Q2) during PAHs biodegradation

The changes of metabolic activity in the individual variants of method treatments are presented in Fig. 5. Analyzes of the results were based on the parameter %Q2, which specifies the percentage of the most metabolically active population. In terms of the differences in %Q2 between the variants after first month of the experiment, it was noted that bioaugmentation did not increase the number of the most metabolically active microorganisms—%Q2 in variants with additional microorganisms was statistically similar to control. Moreover, samples containing PAHs but no additional microorganisms were characterized by the highest number of the most metabolically active cells (6.5 % for soil + PAH and 7 % for soil + PAH + rhamnolipids). The obtained results suggest that the addition of the consortium decreased %Q2 in systems containing PAHs. The differences in the Q2 value between soil inoculated with microorganisms and soil without inoculation resulted from the removal rate of PAHs in the period between the beginning of the experiment and the first month of biodegradation. The addition of microorganisms with a high biodegradation potential and high metabolic activity caused a more rapid removal of PAHs; therefore, upon depletion of the carbon source a decrease of metabolic activity was observed after 1 month of biodegradation in case of inoculated soil, in contrast to non-inoculated soil. In non-inoculated soil, the microorganisms had to undergo an adaptation phase caused by the addition of PAHs, which resulted in the extension of the biodegradation time; hence, the metabolic activity of autochthonic microorganisms during the first month was higher. During the third month, the metabolic activity of microorganisms in all tested soils was similar, which was caused by the complete removal of PAHs. According to Mrozik and Piotrowska-Seget (2010), the competition between indigenous and exogenous microorganisms for limited carbon sources is one of the most important biotic factors affecting the success of bioaugmentation. It is plausible that such competition occurred in the studied case. On the other hand, the higher value of %Q2 observed in systems containing PAHs compared to the variant without PAHs may be associated with the use of hydrocarbons as a carbon source, as confirmed by Silva et al. (2009). Analyzes carried out after the first month of treatment excluded the toxic effects of PAHs on microorganisms in the studied systems—the activity of microorganisms in the samples containing PAHs was not reduced compared to the activity of microorganisms in a systems without PAHs.

A decrease of the %Q2 parameter was observed during the treatment, which means that the number of metabolically active microorganisms decreased with the passage of time. Bento et al. (2005) suggest that this effect may be associated with possible formation of the toxic intermediates during the degradation of higher molecular weight hydrocarbons. However, no differences between control (without hydrocarbons) and soil with PAHs suggest a lack of inhibitory effect of intermediates on microbes. Similar results were observed by Silva et al. (2009). The total PAH content of 600 mg kg^−1^ soil did not contribute to an inhibitory effect toward endogenous and exogenous microorganisms. Thus, the results suggest that the decrease in the %Q2 value was most likely related to the depletion of carbon source and biogenic elements needed for the development of microorganisms. This also resulted in no statistically significant differences between the individual variants after 3, 6, and 12 months of treatment.

## Conclusions

The addition of rhamnolipids and bioaugmentation increased the bioremediation efficiency during the early stage of treatment. The differences between the natural attenuation and other approaches increased with the number of aromatic rings. In addition, the biodegradation rate depended on the structure of PAHs. However, no differences between natural attenuation and other strategies were noticed after 12 months of treatment. This suggests the existence of a sufficient number of contaminant-degrading autochthonous microorganisms and the capacity for natural attenuation in the studied soil. Therefore, in the case of the lack of time pressure, the use of bioaugmentation or rhamnolipids may not be justified.
